# A novel function of artesunate on inhibiting migration and invasion of fibroblast-like synoviocytes from rheumatoid arthritis patients

**DOI:** 10.1186/s13075-019-1935-6

**Published:** 2019-06-24

**Authors:** Jian-Da Ma, Jun Jing, Jun-Wei Wang, Tao Yan, Qian-Hua Li, Ying-Qian Mo, Dong-Hui Zheng, Jin-Long Gao, Ky-Anh Nguyen, Lie Dai

**Affiliations:** 1 Department of Rheumatology, Sun Yat-Sen Memorial Hospital, Sun Yat-Sen University, Guangzhou, 510120 People’s Republic of China; 2 Zhongshan School of Medicine, Sun Yat-Sen University, Guangzhou, People’s Republic of China; 3 Institute of Dental Research, Sydney Dental School, The University of Sydney, Sydney, NSW Australia

**Keywords:** Artesunate, Rheumatoid arthritis, Fibroblast-like synoviocytes, Migration, Invasion

## Abstract

**Introduction:**

Anti-malarial drug artesunate can suppress inflammation and prevent cartilage and bone destruction in collagen-induced arthritis model in rats—suggesting it may be a potent drug for rheumatoid arthritis (RA) therapy. We aimed to investigate its effect on the invasive property of fibroblast-like synoviocytes (FLS) from patients with RA.

**Methods:**

Synovial tissues were obtained by closed needle biopsy from active RA patients, and FLS were isolated and cultured in vitro. RA-FLS were treated with artesunate at various concentrations, while methotrexate or hydroxychloroquine was employed as comparator drugs. Cell viability, proliferation, cell cycle, apoptosis, migration, invasion, and pseudopodium formation of RA-FLS were assessed by CCK-8 assays, EdU staining, Annexin V-FITC/PI staining, transwell assays, or F-actin staining, respectively. Further, relative changes of expressed proteases were analyzed by Proteome profiler human protease array and verified by quantitative real-time PCR (qPCR), Western blot, and ELISA. The expression of signaling molecules of MAPK, NF-κB, AP-1, and PI3K/Akt pathways were measured by qPCR and Western blot. PDK-1 knockdown by specific inhibitor AR-12 or siRNA transfection was used to verify the pharmacological mechanism of artesunate on RA-FLS.

**Results:**

Artesunate significantly inhibited the migration and invasion of RA-FLS in a dose-dependent manner with or without TNF-α stimulation. The effect was mediated through artesunate inhibition of MMP-2 and MMP-9 production, and pre-treatment with exogenous MMP-9 reversed the inhibitory effect of artesunate on RA-FLS invasion. Artesunate had a stronger inhibitory effect on migration and invasion of RA-FLS as well as greater anti-inflammatory effect than those of hydroxychloroquine. Similar inhibitory effect was detected between artesunate and methotrexate, and synergy was observed when combined. Mechanistically, artesunate significantly inhibited PDK-1 expression as well as Akt and RSK2 phosphorylation—in a similar manner to PDK-1-specific inhibitor AR-12 or PDK-1 knockdown by siRNA transfection. This inhibition results in suppression of RA-FLS migration and invasion as well as decreased MMP-2 and MMP-9 expression.

**Conclusions:**

Our study demonstrates artesunate is capable of inhibiting migration and invasion of RA-FLS through suppression of PDK1-induced activation of Akt and RSK2 phosphorylation—suggesting that artesunate may be a potential disease-modifying anti-rheumatic drug for RA.

**Electronic supplementary material:**

The online version of this article (10.1186/s13075-019-1935-6) contains supplementary material, which is available to authorized users.

## Introduction

Rheumatoid arthritis (RA) is a common chronic inflammatory disease characterized by synovitis, leading to the destruction of the articular cartilage and bone [1]. The lining layer of the synovium at the articular borders may become a mass of “pannus” tissue rich in fibroblast-like synoviocytes (FLS) that invade the adjacent articular cartilage. Evidence shows that these activated FLS are critical for the joint destructive process seen in RA [2]. RA-FLS manifest tumor-like properties including increased proliferation, prolonged survival, and apoptosis resistance. They also secret abundant pro-inflammatory cytokines to cause local and systemic inflammation [3]. More importantly, RA-FLS are able to exhibit increased production of matrix metalloproteinases (MMPs) and directly invade the adjacent articular cartilage [4–6]. Thus, targeting RA-FLS represents a potential strategy for RA therapy.

Natural sources such as plant compounds are being developed as anti-rheumatic drugs. *Artemisia annua* L (qinghao) has been recorded as Chinese traditional medicine for the treatment of fever for many centuries, and artemisinin was successfully extracted from the plant by a team of Chinese scientists led by Youyou Tu in 1970s [7]. However, poor biopharmaceutic properties and low bioavailability of artemisinin restricted the clinical application, and its derivatives were developed to increase bioactivity, solubility, and chemical stability. Among the derivatives of artemisinin, artesunate can rapidly distribute and hydrolyze to the biologically active metabolite dihydroartemisinin. Currently, artemisinin and its synthetic derivatives have been recommended by the World Health Organization (WHO) as the first-line therapy for severe malaria with excellent safety profile [8]. Besides anti-malarial properties, artemisinin and its semisynthetic derivatives have been demonstrated to affect various aspects of cellular processes and exhibit potent immunosuppressive activity in several autoimmune disease models, such as systemic lupus erythematosus and RA [9–11].

Previously, artesunate was found to inhibit inflammation, cartilage, and bone destruction in collagen-induced arthritis (CIA) rats; reduce joint inflammation; and attenuate climbing ability and motility in adjuvant-induced arthritis rats as well as prevent the development of arthritis in K/BxN mice [12–14]. This evidence indicated that artesunate may be a potent drug for RA therapy. Recent in vitro studies showed that artesunate could inhibit recombinant human receptor activator of nuclear factor-κB ligand (RANKL)-induced osteoclast differentiation and bone resorption in osteoclast precursors of RAW264.7 cells and mouse bone marrow-derived macrophage cells [15, 16]. Artesunate also decreases the secretion of IL-1β, IL-6, IL-8, and VEGF from TNF-α-stimulated RA-FLS [17, 18]. However, effects of artesunate on other aggressive properties of RA-FLS have not yet been elucidated. Here, we aimed to investigate the potential role of artesunate on inhibition of the tumor-like properties of RA-FLS and its underlying mechanism, particularly in relation to migration and invasion.

In the present study, we found that artesunate suppressed migration and invasion of primary RA-FLS in the absence of inflammatory condition and inhibited the expression of MMP-2 and MMP-9 through suppression of PDK1-induced activation of Akt and RSK2 phosphorylation. We further found that artesunate had stronger inhibitory effect on migration and invasion of RA-FLS as well as greater anti-inflammatory effect than those of hydroxychloroquine (HCQ) at the same safety inhibitory concentration. We also found similar inhibitory profiles between artesunate and methotrexate (MTX), and when combined, they exhibited synergy in inhibiting migration and invasion of RA-FLS. Our study provides evidence for artesunate inhibition of migration and invasion of RA-FLS which implied that artesunate may be a potential disease-modifying anti-rheumatic drug (DMARD) for RA.

## Materials and methods

### Patients and preparation of RA-FLS

Synovial tissues were obtained from 22 patients with active RA by Parker-Pearson needle biopsy [19]. All patients were diagnosed according to the 1987 American College of Rheumatology (ACR) revised classification criteria for RA [20] or the 2010 ACR/the European League against Rheumatology (EULAR) classification criteria for early RA [21], and active disease was defined as disease activity score in 28 joints and four variables including C-reactive protein (DAS28-CRP) ≥ 3.2. This study was approved by the Medical Ethics Committee of Sun Yat-sen Memorial Hospital (SYSEC-KY-KS-034), and all patients signed informed consent.

FLS were isolated by a modified tissue culture method as we described previously [22]. Primary FLS from passages 3~5 were used in our experiments. The morphology of FLS was confirmed under the light microscope and further characterized by flow cytometry using monoclonal antibodies CD90-PE and CD68-Alexa 647 (BD Biosciences, San Diego, CA, USA). Each experiment included isotype-matched control antibodies as methodological controls. The flow cytometric analysis was performed using BD FACS Calibur (BD Biosciences) and data analyzed by BD CellQuest software (BD Biosciences).

### RA-FLS intervention

After serum starvation for 6 h, primary FLS were pre-treated with indicated concentrations of artesunate (Guilin Pharmaceutical Factory, Guilin, China), MTX (Pfizer Pharmaceutical Factory, New York, NY, USA), HCQ (Shanghai Zhongxi Pharmaceutical Factory, Shanghai, China), tumor necrosis factor α (TNF-α, PeproTech, Rocky Hill, NJ, USA), recombinant human MMP-2 or MMP-9 (Sigma-Aldrich, St. Louis, MO, USA), or AR-12 (a specific inhibitor of PDK-1, SelleckChem, Houston, TX, USA) for an indicated time. All above reagents were dissolved in dimethyl sulfoxide (DMSO; Sigma-Aldrich, St. Louis, MO, USA) for storage and then diluted to working concentration with Dulbecco’s modified Eagle medium (DMEM; Thermo Fisher Scientific, Waltham, MA, USA). DMSO diluted with DMEM was taken as the negative control. The final concentration of DMSO was less than 0.1% (*v*/*v*).

### CCK-8 viability assay

The effects of artesunate, MTX, or HCQ as well as the combination of artesunate and MTX on viability of primary RA-FLS were measured by cell counting kit-8 (CCK-8, Dojindo, Japan). Briefly, a total of 5 × 10^3^ cells in logarithmic growth phase in a volume of 100 μl DMEM with 10% FBS were planted in each well of 96-well plate. Cells were treated with indicated concentrations of artesunate, MTX, or HCQ as well as combination of artesunate and MTX, respectively, for an indicated time. Thereafter, cells were added with 10 μM of CCK-8 solution and incubated for 4 h at 37 °C. The absorbance at 450 nm was measured to evaluate cell viability, with a reference wavelength of 630 nm, using an automated microplate reader (Thermo Fisher Scientific, Waltham, MA, USA). The experiment was independently repeated three times from 6 RA patients.

### EdU proliferation assay

The effects of artesunate, MTX, or HCQ on proliferation of primary RA-FLS were measured by 5-ethynyl-2-deoxyuridine (EdU) assay. Cells were treated with indicated concentrations of artesunate, MTX, or HCQ for 24 h and then seeded at a density of 5 × 10^4^ cells/mL into 24-well culture plates. The EdU staining was conducted using a Cell-Light EdU DNA Cell Proliferation Kit (RiboBio, Guangzhou, China) according to the manufacturer’s protocol. Cells were incubated with EdU (1:1000) for 8 h before being harvested by rinsing in PBS, fixed with paraformaldehyde and permeabilized with 0.3% Triton X-100 in PBS for 10 min, and washed. Cells were incubated with Apollo staining reaction solution for 30 min in the dark. Next, cells were nuclear stained with DAPI for 5 min and images were captured using an Olympus laser scanning microscope system. The experiment was independently repeated three times from 6 RA patients.

### Cell-cycle analysis and apoptosis assay

The effects of artesunate, MTX, or HCQ on cell cycle and apoptosis of primary RA-FLS (*n* = 6) were detected by flow cytometry. Briefly, a total of 1 × 10^5^ cells per well were seeded into 6-well culture plates and incubated with artesunate, MTX, or HCQ for 24 h. Cells were then collected and washed three times in PBS. For cell-cycle analysis, the cells were re-suspended in 70% pre-chilled ethanol and fixed at − 20 °C overnight. The cells were washed again with PBS and re-suspended in 200 μL PI/RNase Staining Buffer (BD Pharmingen, San Diego, CA, USA) and incubated at room temperature for 30 min before flow cytometric analysis. For apoptosis assay, the cells were incubated with Annexin V-FITC and PI (BD Biosciences) for 20 min at room temperature. Cell cycle and apoptosis were quantified using the BD FACS Caliber flow cytometer.

### Wound healing assay for horizontal migration

The effects of artesunate, MTX, or HCQ as well as the combination of artesunate and MTX on horizontal migration of primary RA-FLS (*n* = 12) were detected by wound healing assay. Primary RA-FLS (1 × 10^5^) pre-treated with artesunate, MTX, or HCQ with or without TNF-α (100 pg/mL), as well as the combination of artesunate and MTX, for 24 h were seeded into 6-well plates to almost total confluence. To investigate the role of PDK-1 on cell migration, cells were pre-treated for 6 h with PDK-1 inhibitor AR-12 (5 μM). Then, an artificial homogenous wound was created onto the monolayer with a sterile 10-μl tip. After scratching, the culture dishes were washed with serum-free medium. Images of cells migrating into the wound were captured at time 0 and 12 h by an inverted microscope (× 100), and the area of scratch was measured by ImageJ 1.47 analysis system (National Institutes of Health, Bethesda, MD, USA).

### Transwell assays for vertical migration and invasion

The effects of artesunate, MTX, or HCQ as well as the combination of artesunate and MTX on vertical migration and invasion of primary RA-FLS (*n* = 12) were detected using transwell assays. Primary RA-FLS (1 × 10^5^) pre-treated with artesunate, MTX, or HCQ with or without TNF-α (100 pg/mL), as well as the combination of artesunate and MTX for 24 h, were seeded into 6-well plates. To investigate the role of PDK-1 on cell migration and invasion, cells were pre-treated for 6 h with AR-12 (5 μM). Migration assay was performed using transwell chamber with 8-μm pores (Corning Incorporated, Corning, NY, USA). A total of 5 × 10^4^ cells were re-suspended in serum-free medium and seeded in the upper chamber, while the lower chamber was filled with complete medium. After 12 h of incubation, the cells in the upper chamber were carefully removed with a cotton swab, and the cells that had migrated through the membrane to the lower surface were fixed with 4% paraformaldehyde and stained with 0.1% crystal violet. For invasion assay, Matrigel (BD Biosciences) was pre-coated on the membrane of the upper chamber and cells were seeded as migration assay and allowed to invade for 24 h. The cell count was done under the microscope (× 100). Data were presented as the mean of migrated cells in five randomly chosen fields.

### F-actin staining for reorganization of actin cytoskeleton

The effects of artesunate, MTX, or HCQ on modulating actin cytoskeleton reorganization of primary RA-FLS (*n* = 6) were detected by F-actin staining. Primary RA-FLS (1 × 10^3^) were seeded on sterilized glass coverslips in 24-well culture plates. The cells were stimulated with artesunate, MTX, or HCQ for 24 h, and then they were fixed with paraformaldehyde and permeabilized with 0.3% Triton X-100 in PBS. For detection of F-actin, the cells were incubated with phalloidin (Thermo Fisher Scientific Inc., Waltham, MA, USA) overnight. The coverslips were mounted on glass slides with anti-fade mounting media and examined using confocal fluorescence microscopy (LSM 710, Zeiss, Germany).

### Proteome profiler analysis

The relative change of 35 human proteases, including Cathepsins, MMPs (MMP-1~MMP-3 and MMP-7~MMP-13), Kallikreins, ADAMs, DPPIV/CD26, Neprilysin/CD10, Presenilin, Proprotein Convertase 9, Proteinase 3 and uPA/Urokinase, in the culture supernatant derived from primary RA-FLS after artesunate treatment (*n* = 3) were investigated using Proteome Profiler™ human protease array kit (R&D Systems, Minneapolis, MN, USA) following the manufacturer’s protocol. Briefly, primary RA-FLS (1 × 10^5^) were cultured in 6-well plates, then exposed to 60 μM artesunate for 24 h. Culture supernatants were collected, and protein concentration determined by the standard Bradford assay. For each membrane, culture supernatant samples were incubated at room temperature with 15 μL of cytokine and protease detection cocktail for 1 h and incubated overnight at 4 °C. The membranes were then washed with 1× wash buffer and incubated with streptavidin HRP for 30 min. Following another wash, the membranes were incubated with chemi-reagent mix and exposed for 10 min. Spot intensity was quantified using a densitometer, and the average of duplicate spots on the membrane was normalized with the average negative control spots according to the manufacturer’s protocol.

### ELISA detection of cytokine in culture supernatant

The effects of artesunate and MTX as well as the combination of artesunate and MTX on MMPs and tissue inhibitors of metalloproteinase (TIMP) secretion of primary RA-FLS were detected by ELISA. Primary RA-FLS (1 × 10^5^) were seeded in 6-well plates and treated with artesunate and MTX as well as the combination of artesunate and MTX for 24 h with or without TNF-α (100 pg/mL). ELISA assay was performed to verify differentially expressed MMPs screened by Proteome profiler human protease array and their corresponding TIMPs, as well as IL-1β, IL-6, and IL-8 in culture supernatant by using commercial kits (R&D Systems). The culture supernatant was tested in triplicate from 6 RA patients, and cytokine concentrations were determined according to a standard curve from recombinant cytokine.

### Quantitative real-time PCR (qPCR)

Total cellular RNA from primary RA-FLS was extracted using RNAiso Plus reagent (Takara, Otsu, Japan). Complementary DNA (cDNA) samples were synthesized with the reverse-transcription kit (Takara). SYBR green-based qPCR was performed on a Roche LightCycler480 sequence detector system (Roche, Basel, Switzerland). The sequences for the relevant primers are listed in Table 1, and β-actin was used as a quantitative control for RNA levels. All data were calculated using the ^2-ΔΔ^CT method [23]. The experiment was independently repeated three times from 6 RA patients.

**Table 1:** Lists of primer sequences used for quantitative real-time PCR

Target gene	Sequence
MMP-2	Forward: CTCATCGCAGATGCCTGGAA
	Reverse: TTCAGGTAATAGGCACCCTTGAAGA
MMP-9	Forward: TGACAGCGACAAGAAGTG
	Reverse: CAGTGAAGCGGTACATAGG
TIMP-1	Forward: ATCCTGTTGTTGCTGTGGCTGATAG
	Reverse: TGCTGGGTGGTAACTCTTTATTTCA
TIMP-2	Forward: AAACGACATTTATGGCAACCCTATC
	Reverse: ACAGGAGCCGTCACTTCTCTTGATG
PI3K	Forward: CATCACTTCCTCCTGCTCTAT
	Reverse: CAGTTGTTGGCAATCTTCTTC
Akt	Forward: GGACAACCGCCATCCAGACT
	Reverse: GCCAGGGACACCTCCATCTC
PIP2	Forward: AAAACCCTTGGGCTGTTCTT
	Reverse: AATCATGGACGACTCCTTGG
PIP3	Forward: GGGTCAGTGTGACCGAAGAT
	Reverse: GGAAGTCAGAAGTGGGTGGA
PDK-1	Forward: CCAGTCAAGAGCATCAGCAA
	Reverse: AAGTAGTGCAGCCCGGAGTA
RSK2	Forward: GTCTTGGGCCATTACGGGA
	Reverse: GGGGTGCGGAGTGTCTTTT
β-actin	Forward: TGGCACCCAGCACAATGAA
	Reverse: CTAAGTCATAGTCCGCCTAGAAGCA

### Western blot analysis

Proteins in primary RA-FLS lysates from 6 RA patients were separated by SDS-PAGE and transferred to polyvinylidene difluoride membranes. Membranes were blotted with primary antibodies recognizing PIP2, PIP3, PI3K, p-PI3K, PDK-1, Akt (pan), p-Akt (Thr308), RSK2, p-RSK2, p44/p42 MAPK, p38 MAPK, NF-κB p65, NF-κB p-p65, IκBα, p-IκBα, c-fos, c-jun, JNK, p-JNK, MMP-2, MMP-9, TIMP-1, TIMP-2, and GAPDH (all from Cell Signaling Technology, Danvers, MA, USA), respectively, and followed with horseradish peroxidase (HRP)-conjugated secondary antibodies (Cell Signaling Technology). Enhanced chemiluminescence (Millipore, Boston, MA, USA) was applied to detect the targeted proteins. The Western blot membrane was scanned (G: BOX Gel & Blot Imaging Series from Syngene, Cambridge, UK) and densitometry quantified by ImageJ 1.47 analysis system (National Institutes of Health). The intensity of each protein was normalized with GAPDH and represented as a ratio to the control [24].

### Small interfering RNA (siRNA)-mediated knockdown of PDK-1

The siRNA for PDK-1 and negative control (NC) was synthesized by Shanghai Genepharma Co., Ltd. (Shanghai, China). Three independent siRNAs were designed: siRNA#1 (5′-CUUCGGAUCAGUGAAUGCUTT-3′; 5′-AGCAUUCACUGAUCCGAAGTT-3′), siRNA#2 (5′-CAUCCGUUCAAUUGGUACATT-3′; 5′-UGUACCAAUUGAACGGAUGTT-3′, and siRNA#3 (5′-GCGUCUGUGUGAUUUGUAUTT-3′; 5′-AUACAAAUCACACAGACGCTT-3′). The procedures of siRNA transfection were conducted as described previously [23]. Whole cell lysates were extracted to assess knockdown efficiency of PDK-1 in RA-FLS (*n* = 6) by qPCR, and the most silencing efficiency siRNA sequences were used for further experiments. Non-specific NC siRNA were also designed and synthesized. The mock group was defined as that supplemented with the transfection reagent only.

### Statistical analysis

Statistical analyses were performed with SPSS 21.0 statistical software (SPSS Inc., Chicago, USA). Data were presented as mean ± standard deviation (SD) or median and interquartile range (IQR) for continuous variables and presented as frequencies and percentages for categorical variables. Multiple groups of samples were analyzed by one-way ANOVA, and pairwise comparisons were adjusted by the Bonferroni method. Differences were considered statistically significant *P* values < 0.05.

## Results

### Demographic characteristics of the study patients

Twenty-two RA patients were recruited in this study. Among them, 82% were female, the median of age was 46 years (range 21 to 62 years), the median of disease duration was 24 months (range 3 to 240 months), and the median of DAS28-CRP was 5.4 (range 3.8 to 6.7). There were 73% and 73% RA patients with rheumatoid factor positive or anticyclic citrullinated peptide antibody positive, respectively. There were 32% RA patients never taken any corticosteroid or DMARDs previously.

### Artesunate inhibited cytokine production in primary RA-FLS

**Fig. 1:**
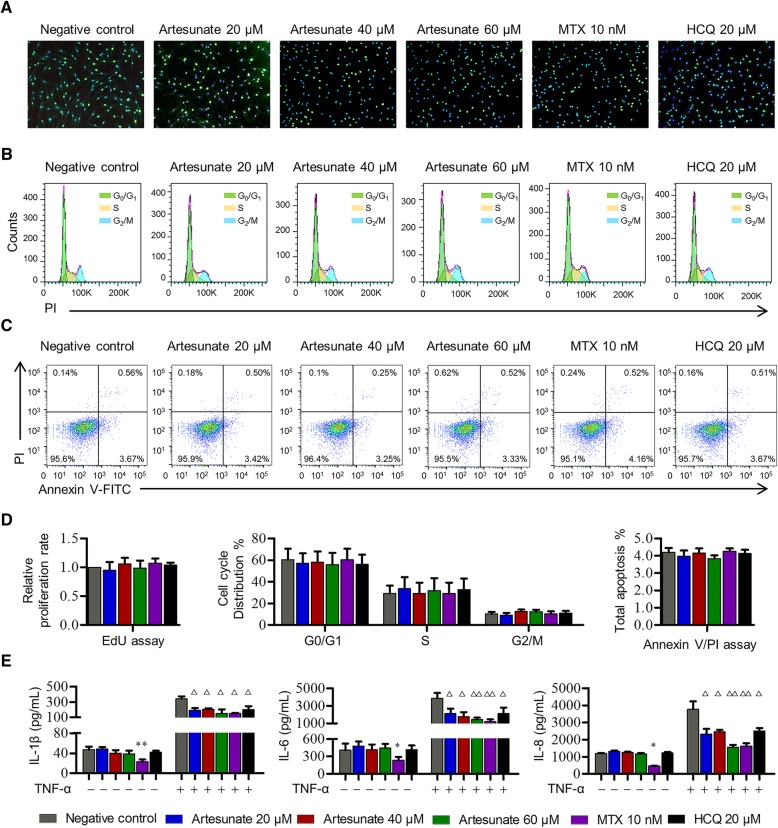
Effects of artesunate on proliferation, cell cycle, apoptosis and cytokine production of primary RA-FLS. RA-FLS were treated with different concentrations of artesunate, MTX (10 nM), or HCQ (20 μM) for 24 h. **a** EdU assays were performed to evaluate the proliferation of RA-FLS. Representative images showed proliferation of RA-FLS labeled with EdU (green) and nuclei stained with DAPI (blue, original magnification, × 100). **b** Cell cycle was detected by flow cytometry with PI staining. **c** Apoptosis was detected by flow cytometry with annexin V-FITC/PI double staining. **d** Bars were representative as effects of artesunate (60 μM), MTX (10 nM), or HCQ (20 μM) on proliferation, cell cycle, and apoptosis of primary RA-FLS measured by EdU, PI, and annexin V-FITC/PI assays, respectively. **e** The levels of IL-1β, IL-6, and IL-8 in culture supernatant of primary RA-FLS with or without 100 pg/mL TNF-α stimulation were measured by ELISA. Data were representative as means ± SD from 6 RA patients. *P < 0.05, **P < 0.01, compared with RA-FLS without TNF-α or artesunate treatment. ^△^*P* < 0.05, ^△△^*P* < 0.01, compared with RA-FLS treated with TNF-α alone

RA-FLS with unique spindle morphology were observed with high expression of surface marker CD90 (93.7% ± 4.26%) but rare expression of surface marker CD68 (0.86% ± 0.35%) by flow cytometry (data not shown). RA-FLS cells were exposed to various concentrations of artesunate and other DMARDs for comparison, including MTX and HCQ. Via cell viability CCK-8 assay, the IC_50_ value of artesunate, MTX, and HCQ after incubation with primary RA-FLS at 24 h were 6891 μM, 181.4 nM, and 5433 μM, respectively. Compared with the untreated group, IC_1_-20 μM, IC_3_-40 μM, IC_5_-60 μM of artesunate, IC_5_-10 nM of MTX, and IC_5_-20 μM of HCQ showed no effect on the proliferation, cell cycle, or apoptosis of RA-FLS (Fig. 1a–d, Additional file 1: Figure S1A). In order to mimic the local inflammatory microenvironment of the synovium in RA, 100 pg/mL of TNF-α was added to stimulate RA-FLS for 24 h. Under this condition, artesunate was able to inhibit IL-1β, IL-6, and IL-8 expression of primary RA-FLS. Artesunate (60 μM) showed similar inhibitory effects to 10 nM MTX (IL-1β: *P* = 0.992, IL-6: *P* = 0.784, IL-8: *P* = 0.107) which were significantly stronger than HCQ (20 μM) at inhibiting IL-6 and IL-8 secretion (IL-1β: *P* = 0.445, IL-6: *P* = 0.034, IL-8: *P* = 0.038, Fig. 1e).

### Artesunate inhibited migration and invasion of primary RA-FLS

**Fig. 2:**
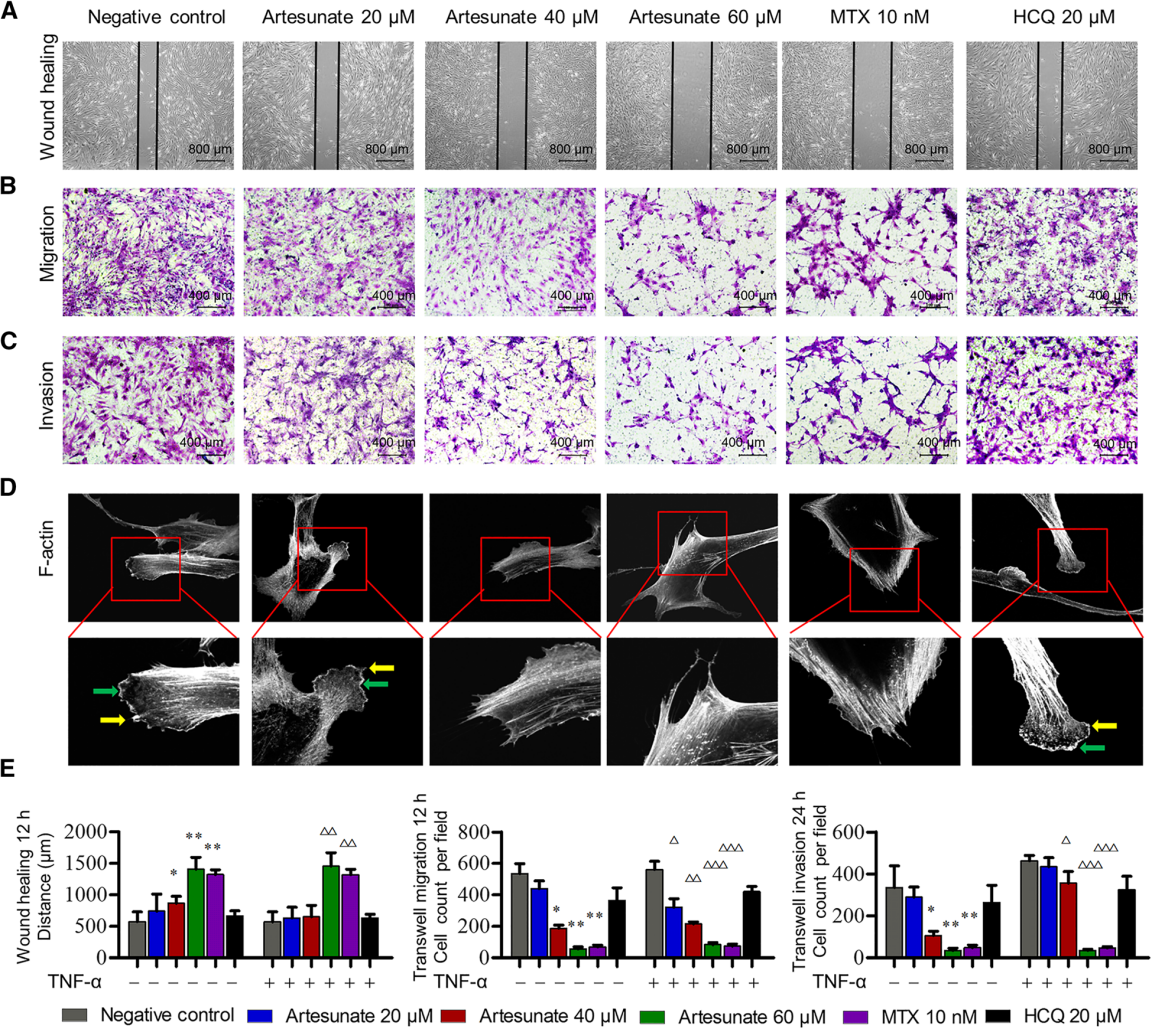
Effects of artesunate on migration and invasion of primary RA-FLS**. a**–**c** Concentrations of artesunate, MTX (10 nM), or HCQ (20 μM) without TNF-α stimulation; wound healing and transwell assays showed the capacity of horizontal migration (**a**), vertical migration (**b**), and invasion (**c**) of primary RA-FLS. **d** F-actin was stained with phalloidin. Representative images were shown (original magnification, × 400 above and × 1000 below). The green arrow indicates lamellipodia formation, and the yellow arrow indicates filopodia formation. **e** Bars were representative as effects indicated concentrations of artesunate, MTX (10 nM), or HCQ (20 μM) on primary RA-FLS migration and invasion measured by wound healing and transwell assays of migration and invasion. Data were representative as means ± SD from 12 RA patients. *P < 0.05, **P < 0.01, compared with RA-FLS without TNF-α or artesunate treatment. ^△^*P* < 0.05, ^△△^*P* < 0.01, ^△△△^*P* < 0.001, compared with RA-FLS treated with TNF-α alone

To explore the effect of artesunate on the aggressive invasive ability, primary RA-FLS were pre-treated with different concentrations of artesunate, MTX, or HCQ for 24 h, respectively. The wound healing and transwell assays of migration and invasion showed that artesunate can inhibit migration and invasion of primary RA-FLS in a dose-dependent manner with or without TNF-α stimulation (Fig. 2a–c). To further confirm the effect of artesunate on RA-FLS migration, modulating actin reorganization was detected by F-actin staining with phalloidin. RA-FLS in the untreated group displayed flat or ruffling lamellipodia and filopodia at their leading edges, whereas RA-FLS treated with artesunate had reduced lamellipodia and filopodia formations (Fig. 2d). Under the same concentration of IC_5_, artesunate (60 μM) showed similar inhibitory effects as MTX (10 nM, all *P* < 0.05 as compared to controls), while HCQ (20 μM) showed no effect on migration and invasion of primary RA-FLS (all *P* > 0.05 as compared to controls, Fig. 2e).

### Artesunate inhibited invasion of primary RA-FLS by attenuating MMP-9 expression

**Fig. 3:**
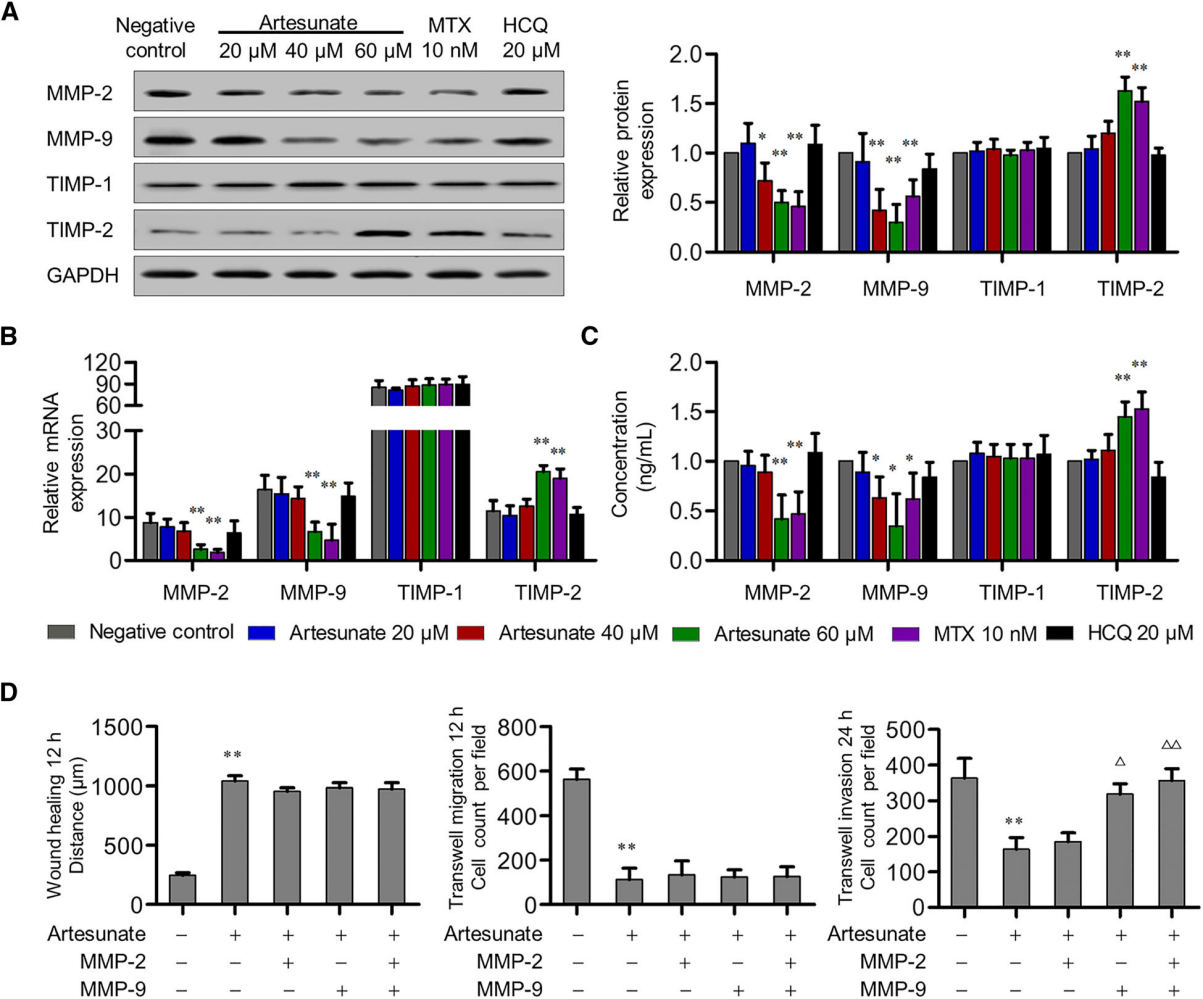
Effects of artesunate on MMP and TIMP expression in primary RA-FLS. **a**–**c** After artesunate, MTX, or HCQ treatment for 24 h, expression of MMP-2, MMP-9, TIMP-1, and TIMP-2 of primary RA-FLS were analyzed by Western blot (**a**), qPCR (**b**), and ELISA (**c**). **a**–**c** shared the same figure key. Each bar represents mean ± SD from 6 RA patients. *P < 0.05, **P < 0.01, compared with RA-FLS without treatment. **d** After treatment of MMP-2 (6 ng/mL) and/or MMP-9 (12 ng/mL) in combination with artesunate (60 μM) for 24 h, wound healing and transwell assays showed the capacity of horizontal migration, vertical migration, and invasion of primary RA-FLS. Bars are representative as means ± SD from 6 RA patients. **P < 0.01, compared with RA-FLS without artesunate treatment. ^△^*P* < 0.05, ^△△^*P* < 0.01 compared with RA-FLS treated with artesunate alone

MMPs play irreplaceable roles in cell invasion. To determine the downstream molecular mediators of artesunate inhibition of invasion, especially MMPs, Proteome Profiler Human Protease Array was used in this study. After 60-μM artesunate treatment for 24 h, the expression of MMP-2 and MMP-9 in the primary RA-FLS culture supernatant were significantly decreased (MMP-2: *P* = 0.021, MMP-9: *P* = 0.006), whereas other MMPs including MMP-1 and MMP-3 remained the same (all *P* > 0.05). Further, qPCR and Western blot verified that both mRNA and protein expression of MMP-2 and MMP-9 in primary RA-FLS were significantly decreased in a dose-dependent manner compared with the untreated group (Fig. 3a–c). This was confirmed again with ELISA showing significantly decreased secreted forms of MMP-2 and MMP-9 in the culture supernatant (△MMP-2, 5.82 ± 0.45 ng/ml; △MMP-9, 11.74 ± 1.30 ng/ml). Meanwhile, artesunate (60 μM) treatment increased the mRNA and protein expression of TIMP-2, but not TIMP-1 (Fig. 3a–c). In contrast to the non-significant effect of HCQ (20 μM) on MMP or TIMP expression, artesunate (60 μM) treatment showed similar inhibition effects to MTX (10 nM) on MMP-2 and MMP-9 expression as well as promotion effect on TIMP-2.

According to the decreased concentration of MMP-2 and MMP-9 in culture supernatant, primary RA-FLS were treated with recombination human MMP-2 (6 ng/ml) and/or MMP-9 (12 ng/ml) to restore to the levels detected in control cells, and in combination with artesunate (60 μM) for 24 h. The results showed that only supplemented MMP-9 could reverse the inhibitory effect of artesunate on invasion of primary RA-FLS, but not migration, whereas MMP-2 alone could not (Fig. 3d). These results indicated that artesunate might inhibit invasion of primary RA-FLS by attenuating MMP-9 expression.

### Synergistic effect of artesunate with MTX on inhibiting migration and invasion of primary RA-FLS

**Fig. 4:**
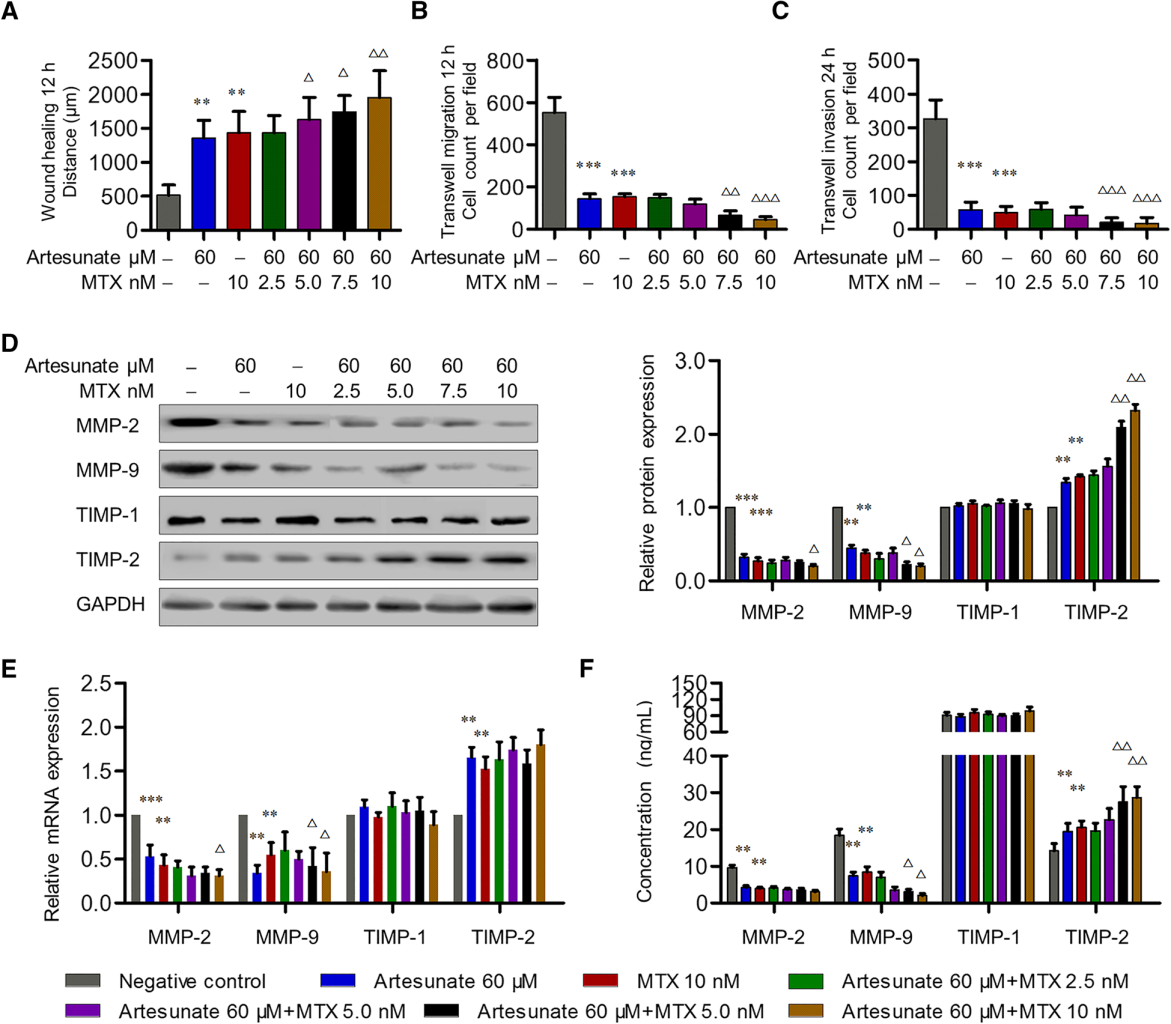
Effects of combined treatment with artesunate and MTX on primary RA-FLS. **a**–**c** After combined treatment with artesunate and MTX for 24 h, wound healing and transwell assays showed the capacity of horizontal migration (**a**), vertical migration (**b**), and invasion (**c**) of primary RA-FLS. **d**–**f** Effects of combined treatment with artesunate and MTX on the expression of MMP-2, MMP-9, TIMP-1, and TIMP-2 in primary RA-FLS were measured by Western blot (**d**), qPCR (**e**), and ELISA (**f**). **d**–**f** shared the same figure key. Each bar represents mean ± SD from 6 RA patients. *P < 0.05, **P < 0.01, ***P < 0.001, compared with RA-FLS without artesunate treatment. ^△^*P* < 0.05, ^△△^*P* < 0.01, compared with RA-FLS treatment with MTX (10 nM)

MTX has been considered as an anchor drug in RA treatment. However, it potentially has serious side effects which are mostly dose-dependent. To explore the synergistic effect of artesunate with MTX on inhibiting aggressive ability of RA-FLS, primary RA-FLS were pre-treated with artesunate (60 μM) and different concentrations of MTX for 24 h, respectively. Compared with the untreated group, combination of artesunate and MTX (2.5–10 nM) showed no significant effect on proliferation of primary RA-FLS for 72 h (Additional file 1: Figure S1B). For wound healing and transwell assays of migration and invasion of primary RA-FLS, the combination of artesunate and MTX (2.5–5 nM) showed similar inhibitory effects on migration and invasion as well as MMP-2 and MMP-9 suppression when compared with MTX (10 nM) alone, while the combination of artesunate and higher concentrations of MTX (7.5–10 nM) had significantly stronger inhibitory effect on migration and invasion than MTX (10 nM) alone (Fig. 4). These results indicated the synergistic effect of artesunate with MTX on inhibiting migration and invasion of primary RA-FLS, which implied that the combination of artesunate may reduce the dosage of MTX for the same efficacy but more safety.

### Artesunate inhibited RA-FLS migration and invasion through PDK-1 pathway

**Fig. 5:**
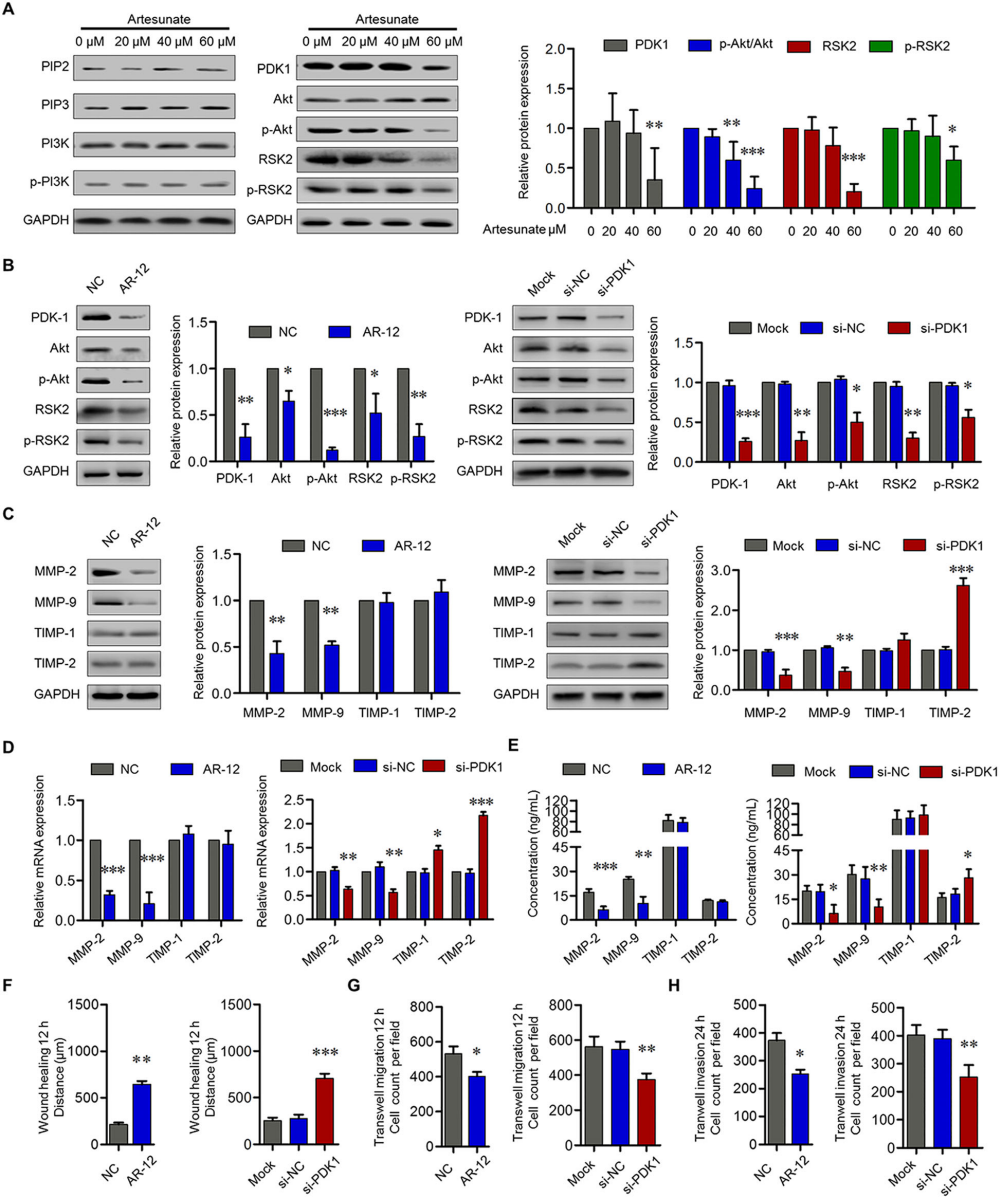
Effects of artesunate on PI3K/Akt/RSK2 pathway in primary RA-FLS. **a** Effects of artesunate (0–60 μM) on the expression of PIP2, PIP3, PI3K, p-PI3K, PDK-1, Akt, p-Akt, RSK2, and p-RSK2 were measured by Western blot. **b** Effects of AR-12 (5 μM) or si-PDK-1 on the expression of PDK-1, Akt, p-Akt, RSK, and p-RSK in primary RA-FLS were measured by Western blot. **c** Effects of AR-12 (5 μM) or si-PDK-1 on the expression of MMP-2, MMP-9, TIMP-1, and TIMP-2 in primary RA-FLS were measured by Western blot (**c**), qPCR (**d**), and ELISA (**e**). **f**–**h** After treatment with AR-12 (5 μM) or si-PDK-1, wound healing and transwell assays showed the capacity of horizontal migration (**f**), vertical migration (**g**), and invasion (**h**) of primary RA-FLS. Each bar represents mean ± SD from 6 RA patients. *P < 0.05, **P < 0.01, ***P < 0.001, compared with RA-FLS without treatment or si-NC group

MMP gene expression in FLS is mainly regulated at the transcriptional level. The key promoters in MMP genes contain AP-1 and NF-κB binding sites, and both MAPK and PI3K/Akt pathways were also involved in the regulation of MMP expression [25]. To explore the effects of artesunate on the PI3K/Akt, MAPK, AP-1, or NF-κB signaling pathways, primary RA-FLS cells were pre-treated with different concentrations of artesunate for 24 h before being assessed by Western blots against specific targets. The results showed that artesunate (60 μM) did not alter the mRNA and protein expression of PI3K and Akt in primary RA-FLS, nor levels of p44/42 MAPK, p38 MAPK, JNK, and p-JNK from MAPK pathway; IKK-α, IKK-β, IκB, p-IκBα, NF-κB p65, and NF-κB p-p65 from NF-κB pathway; or c-fos and c-jun from AP-1 pathway (data not shown). However, phosphorylation of Akt and RSK2 were significantly inhibited by artesunate in a dose-dependent manner. Probing for regulators of Akt and RSK2 phosphorylation showed that artesunate significantly inhibited PDK-1 expression, but not PIP2 and PIP3, which implied that artesunate may inhibit Akt and RSK2 activation through PDK-1 suppression (Fig. 5a).

To verify whether artesunate inhibits RA-FLS migration and invasion through PDK-1, primary RA-FLS were pre-treated with AR-12 (specific inhibitor of PDK-1, 5 μM) for 6 h or transfected with siRNA-PDK-1 for 48 h. The results showed lowered expression of Akt, p-Akt, RSK2, and p-RSK2 (Fig. 5b). Inhibition of PDK-1 by AR-12 or siRNA significantly suppressed the expression of MMP-2 and MMP-9, but only transfection with siRNA-PDK-1 increased the expression of TIMP-2 (Fig. 5c–e). Lastly, PDK-1 inhibitor AR-12 or PDK-1 knockdown by siRNA significantly suppressed wound healing as well as inhibited migration and invasion of RA-FLS (Fig. 5f–h), which implied that artesunate inhibited RA-FLS migration and invasion through blocking PDK-1 and its downstream pathways.

## Discussion

Benefiting from advances in our understanding of RA pathogenesis, numerous efficacious agents for RA treatment are presently available with the application of biological agents and targeted synthetic DMARDs (tsDMARDs) being regarded as a milestone in RA treatment [26]. However, clinical trials of approved biological agents for RA including TNF-α inhibitors, IL-6 receptor inhibitor, T cell co-stimulation blocker, B cell eliminator, and intracellular signal inhibitors of JAK pathway showed similar response with limited efficacy of 30~ 40% in ACR70 improvement rates, whereas therapeutic failure appeared in the blockage of other targets such as IL-1, IL-12, IL-17, IL-20, IL-21, IL-23, anti-CD4, anti-BAFF, and inhibitors of intracellular signal molecular of p38-MAPK and SYK [1]. RA-FLS play critical roles not only on inflammation, but also on cartilage degradation and subsequent bone destruction by their tumor-like properties of migration and invasion. Targeting RA-FLS is more important for RA therapy as a DMARD. As expensive cost and the increasing risk of serious infection and skin cancers limited the application of biological agents and tsDMARDs in real-world clinical practice especially in developing countries, we focused on drugs extracted from natural sources and found a novel function of artemisinin derivative artesunate on inhibition of tumor-like properties of RA-FLS at a safe concentration of the drug.

Exogenous TNF-α is widely used to mimic RA inflammatory microenvironment in vitro study. Artesunate has been reported to decrease the secretion of IL-1β, IL-6, IL-8, and VEGF from TNF-α-stimulated RA-FLS in a dose-dependent manner at concentrations of 5~ 20 μM [17, 18]. However, cytokines can promote migration and invasion of RA-FLS as well as their pro-inflammatory effect. In this study, we found that artesunate inhibited migration and invasion of TNF-α-stimulated RA-FLS as well as inhibiting their secretion of IL-1β, IL-6, and IL-8 at concentrations of 20~ 40 μM. Further experiments without exogenous TNF-α-stimulation showed that surprisingly, although no significant effect on pro-inflammatory cytokines were seen, artesunate at IC_5_ significantly inhibited migration and invasion of primary RA-FLS which was similar to the effect of MTX at IC_5_. These data indicated that artesunate directly inhibited migration and invasion of RA-FLS in the absence of inflammatory condition as well as suppress their pro-inflammatory effect under inflammatory environment. As artesunate was also reported to inhibit RANKL-induced osteoclast differentiation and bone resorption by RAW264.7 cells through suppression of NFATc1 activation and by primary bone marrow-derived macrophage cells through suppression of the NF-κB signaling pathway, it could be a potential DMARD for RA which may both inhibit inflammation and prevent joint destruction progression especially for patients with low inflammation [15, 16].

From the results of Proteome Profiler human array which contained 35 human proteases in different categories related to matrix degradation, we found artesunate inhibited the expression of MMP-2 and MMP-9 secreted from primary RA-FLS and promoted TIMP-2 expression. TIMPs belong to a gene family consisting of four different members of TIMP-1, TIMP-2, TIMP-3, and TIMP-4. TIMP-1 and TIMP-2 are unique in that they make the complexes with MMP-9 and MMP-2 by binding in a 1:1 M ratio (i.e., the MMP-9/TIMP-1 and MMP-2/TIMP-2 complexes) and inhibit the activation of MMP-2 and MMP-9 [27]. Our data showed supplementation of MMP-9 successfully reversed the inhibitory effect of artesunate on invasion of primary RA-FLS which confirmed that artesunate inhibits RA-FLS invasion through suppression of MMP-9 expression. However, supplementation with MMP-2 could not reverse the inhibitory effect of artesunate on invasion of primary RA-FLS. As TIMP-2 is unique in making complex with proMMP-2 and inhibits the activity of MMP-2, our results implied that artesunate likely inhibits RA-FLS invasion through upregulation of TIMP-2 as well as downregulation of MMP-2 expression.

**Fig. 6:**
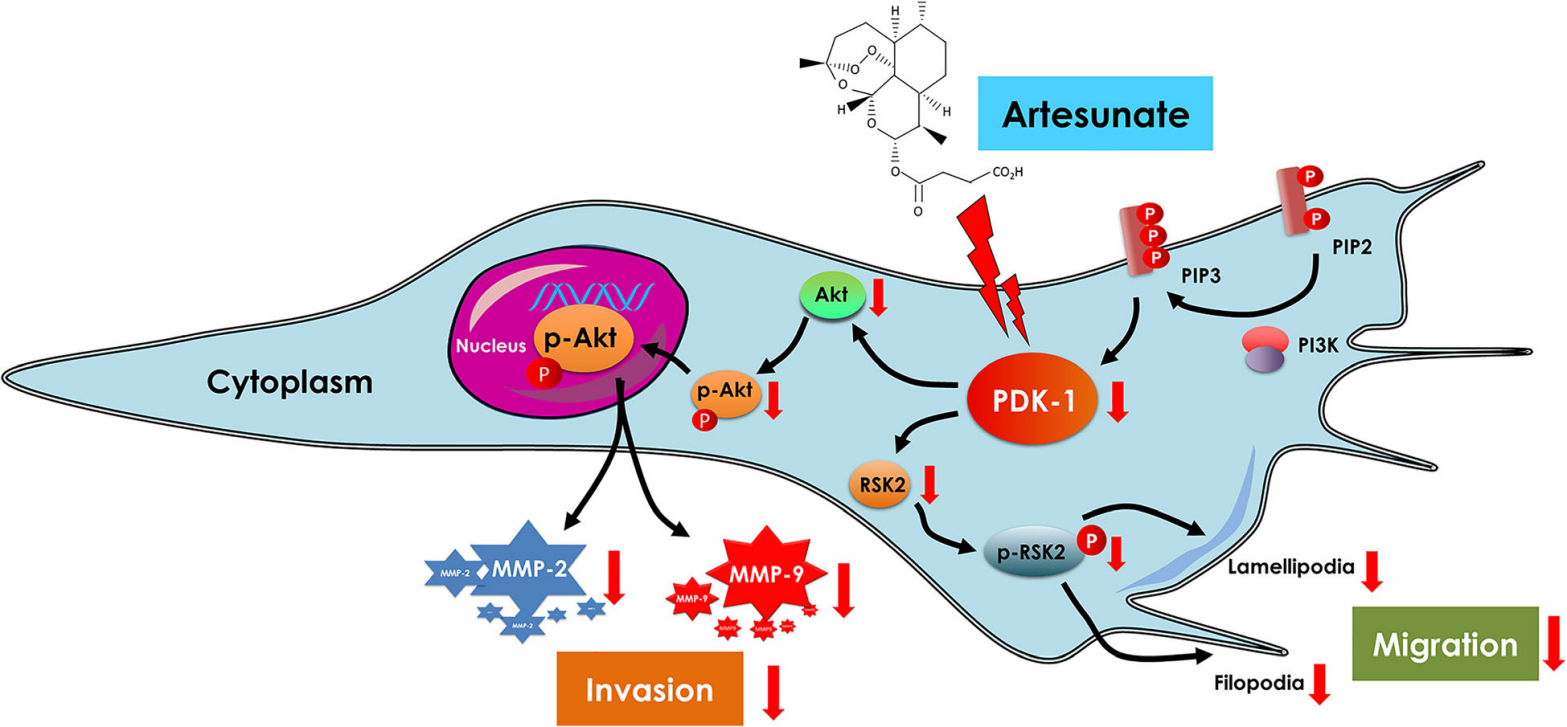
Proposed mechanism of artesunate on inhibition of RA-FLS migration and invasion

Several signaling pathways have been reported to be important to RA-FLS phenotype, including inflammation-related pathways of MAPK, NF-κB and AP-1, or integrin/galectin triggered PI3K/Akt pathway [28]. Our data showed artesunate inhibited migration and invasion of primary RA-FLS in the absence of TNF-α-stimulation through suppression of PDK-1-induced activation of Akt. In contrast, no effect on NF-κB, MAPK, or AP-1 pathways was detected. The PI3K/Akt pathway plays a pivotal role in regulating cellular functions, including cell growth, proliferation, differentiation, motility, survival, and intracellular trafficking, as well as controlling the secretion of pro-inflammation cytokines and matrix-degrading enzymes and chemokines in RA-FLS [29]. PDK-1 lies directly downstream of PI3K, which is in turn, activated by the membrane recruitment by the second messenger PIP3 and serves as a master regulator of the AGC family of protein kinases such as Akt [30]. Subsequently, p-Akt is a downstream effector in this pathway which has been shown to regulate the transcription of MMPs such as MMP-2 and MMP-9 (Fig. 6) [31]. RSK2 is another downstream effector of PDK-1 which can activate RhoA GTPase and promote actin cytoskeleton remodeling, leading to pseudopodia formation and migration [32]. In our study, a specific inhibitor of PDK-1 inhibited migration and invasion of RA-FLS as well as suppressed MMP-2 and MMP-9 expression, but they could not alter the TIMP-1 and TIMP-2 expression. This indicated that artesunate likely inhibits migration and invasion of RA-FLS as well as MMP-2 and MMP-9 expression through suppression of the PDK-1 pathway.

Hydroxychloroquine has been accepted as DMARDs for RA by American College of Rheumatology (ACR) [33]. The mechanism of action of HCQ is proposed to be related to interference with lysosomal activity, inhibition of antigen presentation, and toll-like receptor signaling [34]. However, HCQ showed mild anti-rheumatic activity and may not retard the progression of joint destruction, especially in comparison with other agents. HCQ had not been listed as DMARDs in the European League against Rheumatology (EULAR) until recently, and it was found to exert positive effects on lipid and glucose metabolism and reduction of cardiovascular risk [35, 36]. Our data showed that artesunate had a stronger inhibitory effect on migration and invasion of RA-FLS as well as a greater anti-inflammatory effect than HCQ. Further, artemisinin has been reported to regulate other cellular biochemical processes such as inhibiting triacylglycerol synthesis; thus, it could potentially reduce cardiovascular risk as for HCQ [37]. These findings implied that artesunate may be a more efficacious DMARD compared to HCQ.

Methotrexate is the anchor drug for RA treatment and its probable mechanism includes antagonism of folate-dependent processes, stimulation of adenosine signaling, and inhibition of methyl-donor production, resulting in downregulation of adhesion molecule, pro-inflammation cytokines, and MMP expression [38]. However, MTX is also a cytotoxic drug causing various side effects including fatal adverse effects which is closely related to its dosage. Previous studies have demonstrated that 100 nM MTX inhibited proliferation of RA-FLS and MTX at this concentration could also induce apoptosis of RA-FLS [39]. Considering the different regulation pathways between artesunate and MTX, combination therapy may be a potential multi-target strategy to improve efficacy and reduce adverse effects in RA. Our data showed that artesunate had a synergistic effect with MTX on inhibiting migration and invasion of primary RA-FLS and a combination of artesunate may reduce the dosage of MTX required to achieve the of same clinical efficacy—this will be the focus for our future study.

## Conclusions

In summary, this study provides evidence for artesunate inhibition of migration and invasion of RA-FLS through suppression of PDK1-induced activation of Akt and RSK2 phosphorylation (Fig. 6). Coupled with synergistic effect in combination with MTX, artesunate may represent a potential new DMARD for RA treatment.

## Additional file

Additional file 1: **Figure S1.** Effects of artesunate, MTX, and HCQ on primary RA-FLS viability. (A) The effect of artesunate, MTX, or HCQ on viability of primary RA-FLS was measured by CCK-8 assays at 6, 12, 24, 36, 48, and 72 h. Data were representative as means ± SD from 6 RA patients. (B) Effects of artesunate (60 μM), MTX (10 nM), or combined treatment with artesunate (60 μM) and MTX (2.5~10 nM) on viability of primary RA-FLS were measured by CCK-8 assays at 6, 12, 24, 36, 48, and 72 h. Data were representative as means ± SD from 6 RA patients. (DOCX 489 kb)

## Data Availability

The datasets used and/or analyzed during the current study are available from the corresponding author on reasonable request.

## Abbreviations

ACR: American College of Rheumatology
CCK-8: Cell counting kit-8
DAS28-CRP: Disease activity score 28-joint with four variables including CRP
DMARDs: Disease-modifying anti-rheumatic drugs
EULAR: European League against Rheumatology
FLS: Fibroblast-like synoviocytes
HCQ: Hydroxychloroquine
MMP: Matrix metalloproteinase
MTX: Methotrexate
qPCR: Quantitative real-time PCR
RA: Rheumatoid arthritis
TIMPs: Tissue inhibitors of metalloproteinases

## References

[1] Smolen JS, Aletaha D, McInnes IB (2016). Rheumatoid arthritis. Lancet..

[2] Orr C, Vieira-Sousa E, Boyle DL, Buch MH, Buckley CD, Cañete JD (2017). Synovial tissue research: a state-of-the-art review. Nat Rev Rheumatol.

[3] Korb-Pap A, Bertrand J, Sherwood J, Pap T (2016). Stable activation of fibroblasts in rheumatic arthritis-causes and consequences. Rheumatology (Oxford).

[4] Ma JD, Zhou JJ, Zheng DH, Chen LF, Mo YQ, Wei XN (2014). Serum matrix metalloproteinase-3 as a noninvasive biomarker of histological synovitis for diagnosis of rheumatoid arthritis. Mediat Inflamm.

[5] Ma JD, Wei XN, Zheng DH, Mo YQ, Chen LF, Zhang X (2015). Continuously elevated serum matrix metalloproteinase-3 for 3 ~ 6 months predict one-year radiographic progression in rheumatoid arthritis: a prospective cohort study. Arthritis Res Ther.

[6] Xue M, McKelvey K, Shen K, Minhas N, March L, Park SY (2014). Endogenous MMP-9 and not MMP-2 promotes rheumatoid synovial fibroblast survival, inflammation and cartilage degradation. Rheumatology (Oxford).

[7] Tu Y (2011). The discovery of artemisinin (qinghaosu) and gifts from Chinese medicine. Nat Med.

[8] Organization, W. H (2015). Guidelines for the treatment of malaria.

[9] An J, Minie M, Sasaki T, Woodward JJ, Elkon KB (2017). Antimalarial drugs as immune modulators: new mechanisms for old drugs. Annu Rev Med.

[10] Feng X, Chen W, Xiao L, Gu F, Huang J, Tsao BP (2017). Artesunate inhibits type I interferon-induced production of macrophage migration inhibitory factor in patients with systemic lupus erythematosus. Lupus..

[11] Lin ZM, Yang XQ, Zhu FH, He SJ, Tang W, Zuo JP (2016). Artemisinin analogue SM934 attenuate collagen-induced arthritis by suppressing T follicular helper cells and T helper 17 cells. Sci Rep.

[12] Li Y, Wang S, Wang Y, Zhou C, Chen G, Shen W (2013). Inhibitory effect of the antimalarial agent artesunate on collagen-induced arthritis in rats through nuclear factor kappa B and mitogen-activated protein kinase signaling pathway. Transl Res.

[13] Guruprasad B, Chaudhary P, Choedon T, Kumar VL (2015). Artesunate ameliorates functional limitations in Freund’s complete adjuvant-induced monoarthritis in rat by maintaining oxidative homeostasis and inhibiting COX-2 expression. Inflammation..

[14] Hou L, Block KE, Huang H (2014). Artesunate abolishes germinal center B cells and inhibits autoimmune arthritis. PLoS One.

[15] Zeng X, Zhang Y, Wang S, Wang K, Tao L, Zou M (2017). Artesunate suppresses RANKL-induced osteoclastogenesis through inhibition of PLCγ1-Ca2+-NFATc1 signaling pathway and prevents ovariectomy-induced bone loss. Biochem Pharmacol.

[16] Wei CM, Liu Q, Song FM, Lin XX, Su YJ, Xu J (2018). Artesunate inhibits RANKL-induced osteoclastogenesis and bone resorption in vitro and prevents LPS-induced bone loss in vivo. J Cell Physiol.

[17] Xu H, He Y, Yang X, Liang L, Zhan Z, Ye Y (2007). Anti-malarial agent artesunate inhibits TNF-alpha-induced production of proinflammatory cytokines via inhibition of NF-kappaB and PI3 kinase/Akt signal pathway in human rheumatoid arthritis fibroblast-like synoviocytes. Rheumatology (Oxford).

[18] He Y, Fan J, Lin H, Yang X, Ye Y, Liang L (2011). The anti-malaria agent artesunate inhibits expression of vascular endothelial growth factor and hypoxia-inducible factor-1α in human rheumatoid arthritis fibroblast-like synoviocyte. Rheumatol Int.

[19] Schumacher HR, Kulka JP (1972). Needle biopsy of the synovial membrane--experience with the Parker-Pearson technic. N Engl J Med.

[20] Arnett FC, Edworthy SM, Bloch DA, McShane DJ, Fries JF, Cooper NS (1988). The American Rheumatism Association 1987 revised criteria for the classification of rheumatoid arthritis. Arthritis Rheum.

[21] Aletaha D, Neogi T, Silman AJ, Funovits J, Felson DT, Bingham CO (2010). 2010 rheumatoid arthritis classification criteria: an American College of Rheumatology/European League Against Rheumatism collaborative initiative. Ann Rheum Dis.

[22] Zhou JJ, Ma JD, Mo YQ, Zheng DH, Chen LF, Wei XN (2014). Down-regulating peroxisome proliferator-activated receptor-gamma coactivator-1 beta alleviates the proinflammatory effect of rheumatoid arthritis fibroblast-like synoviocytes through inhibiting extracellular signal-regulated kinase, p38 and nuclear factor-kappaB activation. Arthritis Res Ther..

[23] Chen X, Xie R, Gu P, Huang M, Han J, Dong W (2019). Long noncoding RNA LBCS inhibits self-renewal and chemoresistance of bladder cancer stem cells through epigenetic silencing of SOX2. Clin Cancer Res.

[24] Gu P, Chen X, Xie R, Han J, Xie W, Wang B (2017). lncRNA HOXD-AS1 regulates proliferation and chemo-resistance of castration-resistant prostate cancer via recruiting WDR5. Mol Ther.

[25] Bartok B, Firestein GS (2010). Fibroblast-like synoviocytes: key effector cells in rheumatoid arthritis. Immunol Rev.

[26] Noack M, Miossec P (2017). Selected cytokine pathways in rheumatoid arthritis. Semin Immunopathol.

[27] Khokha R, Murthy A, Weiss A (2013). Metalloproteinases and their natural inhibitors in inflammation and immunity. Nat Rev Immunol.

[28] Tanner MR, Pennington MW, Laragione T, Gulko PS, Beeton C (2017). KCa1.1 channels regulate β1-integrin function and cell adhesion in rheumatoid arthritis fibroblast-like synoviocytes. FASEB J.

[29] Hayer S, Pundt N, Peters MA, Wunrau C, Kühnel I, Neugebauer K (2009). PI3Kgamma regulates cartilage damage in chronic inflammatory arthritis. FASEB J.

[30] Gagliardi PA, di Blasio L, Primo L (2015). PDK1: a signaling hub for cell migration and tumor invasion. Biochim Biophys Acta.

[31] Chao W, Deng JS, Huang SS, Li PY, Liang YC, Huang GJ (2017). 3, 4-dihydroxybenzalacetone attenuates lipopolysaccharide-induced inflammation in acute lung injury via down-regulation of MMP-2 and MMP-9 activities through suppressing ROS-mediated MAPK and PI3K/AKT signaling pathways. Int Immunopharmacol.

[32] Saag KG, Teng GG, Patkar NM, Anuntiyo J, Finney C, Curtis JR (2008). American College of Rheumatology 2008 recommendations for the use of nonbiologic and biologic disease-modifying antirheumatic drugs in rheumatoid arthritis. Arthritis Rheum.

[33] Shi GX, Yang WS, Jin L, Matter ML, Ramos JW (2018). RSK2 drives cell motility by serine phosphorylation of LARG and activation of Rho GTPases. Proc Natl Acad Sci U S A.

[34] Rempenault C, Combe B, Barnetche T, Gaujoux-Viala C, Lukas C, Morel J (2018). Metabolic and cardiovascular benefits of hydroxychloroquine in patients with rheumatoid arthritis: a systematic review and meta-analysis. Ann Rheum Dis.

[35] Smolen JS, Landewé R, Breedveld FC, Buch M, Burmester G, Dougados M (2014). EULAR recommendations for the management of rheumatoid arthritis with synthetic and biological disease-modifying antirheumatic drugs: 2013 update. Ann Rheum Dis.

[36] Smolen JS, Landewé R, Bijlsma J, Burmester G, Chatzidionysiou K, Dougados M (2017). EULAR recommendations for the management of rheumatoid arthritis with synthetic and biological disease-modifying antirheumatic drugs: 2016 update. Ann Rheum Dis.

[37] Zheng H, Colvin CJ, Johnson BK, Kirchhoff PD, Wilson M, Jorgensen-Muga K (2017). Inhibitors of Mycobacterium tuberculosis DosRST signaling and persistence. Nat Chem Biol.

[38] Brown PM, Pratt AG, Isaacs JD (2016). Mechanism of action of methotrexate in rheumatoid arthritis, and the search for biomarkers. Nat Rev Rheumatol.

[39] Xu K, Cai YS, Lu SM, Li XL, Liu L, Li Z (2015). Autophagy induction contributes to the resistance to methotrexate treatment in rheumatoid arthritis fibroblast-like synovial cells through high mobility group box chromosomal protein 1. Arthritis Res Ther..

