# Supplementary material for: A novel function of artesunate on inhibiting migration and invasion of fibroblast-like synoviocytes from rheumatoid arthritis patients
Source: Arthritis Res Ther. 2019 Jun 24;21:153. doi: 10.1186/s13075-019-1935-6 (PMC6591920; doi:10.1186/s13075-019-1935-6)
Supplement: Supplementary file 1 — Figure S1. Effects of artesunate, MTX, and HCQ on primary RA-FLS viability. (A) The effect of artesunate, MTX, or HCQ on viability of primary RA-FLS was measured by CCK-8 assays at 6, 12, 24, 36, 48, and 72 h. Data were representative as means ± SD from 6 RA patients. (B) Effects of artesunate (60 μM), MTX (10 nM), or combined treatment with artesunate (60 μM) and MTX (2.5~10 nM) on viability of primary RA-FLS were measured by CCK-8 assays at 6, 12, 24, 36, 48, and 72 h. Data were representative as means ± SD from 6 RA patients. (DOCX 489 kb) [file 13075_2019_1935_MOESM1_ESM.docx]

**Additional file 1**


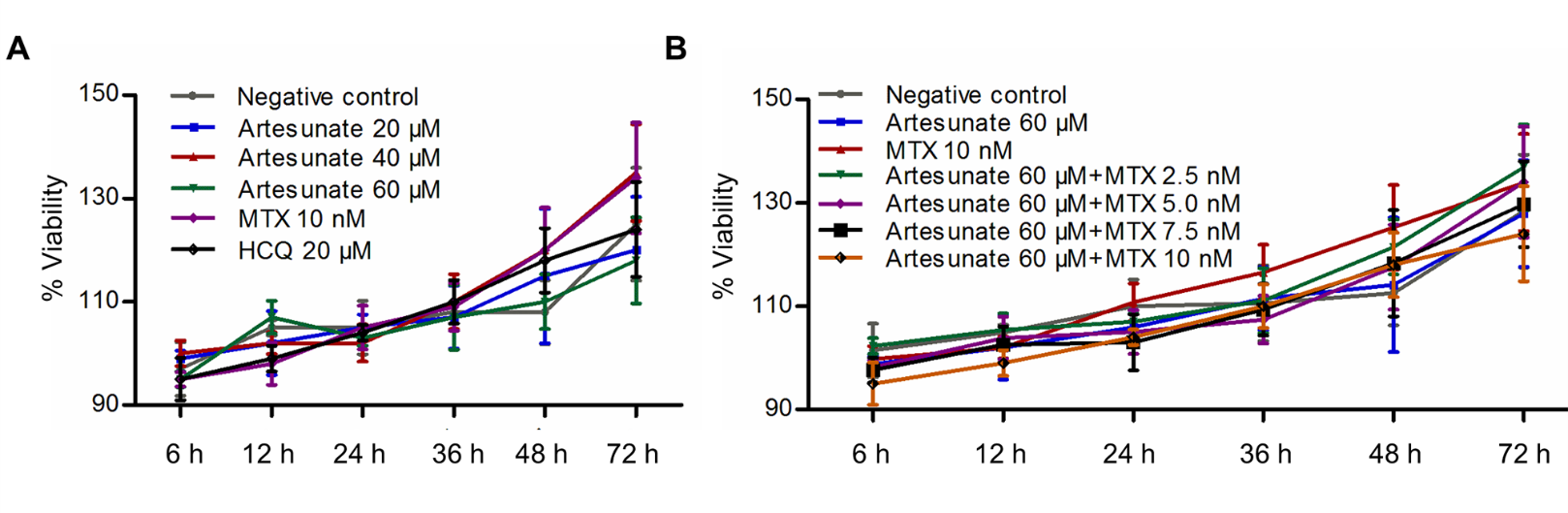


**Figure S1 Effects of artesunate, MTX and HCQ on primary RA-FLS viability.** **(A)** The effect of artesunate, MTX or HCQ on viability of primary RA-FLS were measured by CCK-8 assays at 6, 12, 24, 36, 48 and 72 hours. Data were representative as means ± SD from 6 RA patients. **(B)** Effects of artesunate (60 μM), MTX (10 nM) or combined treatment with artesunate (60 μM) and MTX (2.5~10 nM) on viability of primary RA-FLS were measured by CCK-8 assays at 6, 12, 24, 36, 48 and 72 hours. Data were representative as means ± SD from 6 RA patients.
